# Lumbar Spine Orientation Affects Compressive Fracture Outcome

**DOI:** 10.1007/s10439-024-03604-y

**Published:** 2024-10-25

**Authors:** Rachel Cutlan, Muhammad Khokhar, Nader Shammout, Alok S. Shah, Lance Frazer, Narayan Yoganandan, Barry S. Shender, James Sheehy, Glenn Paskoff, Daniel Nicolella, Timothy Bentley, Saman Shabani, Brian D. Stemper

**Affiliations:** 1 Department of Biomedical Engineering, Marquette University and Medical College of Wisconsin, Milwaukee, WI USA; 2 Department of Neurosurgery, Medical College of Wisconsin, Milwaukee, WI USA; 3 Southwest Research Institute, San Antonio, TX USA; 4 Zablocki Veterans Affairs Medical Center, Milwaukee, WI USA; 5 Naval Air Warfare Center Aircraft Division, Patuxent River, MD USA; 6 Office of Naval Research, Arlington, VA USA

**Keywords:** Biomechanics, Lumbar lordosis, Vertebral body fracture, Axial loading

## Abstract

**Purpose:**

Understanding how spinal orientation affects injury outcome is essential to understand lumbar injury biomechanics associated with high-rate vertical loading.

**Methods:**

Whole-column human lumbar spines (T12–L5) were dynamically loaded using a drop tower to simulate peak axial forces associated with high-speed aircraft ejections and helicopter crashes. Spines were allowed to maintain natural lordotic curvature for loading, resulting in a range of orientations. Pre-test X-rays were used to quantify specimen orientation at the time of loading. Primary fracture types were identified (wedge, *n* = 6; burst, *n* = 4; hyperextension, *n* = 4) and compared for loading parameters and lumbar orientation.

**Results:**

Fracture type was dependent on peak acceleration, bending moment, Cobb angle, sagittal spinal tilt, and location of the applied load.

**Conclusions:**

Lumbar spine orientation under high-rate axial acceleration affected the resulting fracture type. Analysis of pre-test X-rays revealed that spines that sustained wedge and burst fractures were oriented straighter at the time of loading. The load was applied centrally to T12 in spines with burst fractures, and anteriorly to T12 in spines with wedge fractures. Spines that sustained hyperextension fracture had lower peak accelerations, larger Cobb angles at the time of loading, and sustained larger extension moments. Fracture presentation is an important and understudied factor that influences biomechanical stability, clinical course, and long-term patient outcomes.

## Introduction

Lumbar spine injuries are common in military members [25, 50]. Helicopter crashes, fixed wing high-speed aircraft ejections, and under-body blasts all involve high-rate vertical acceleration. Vertical acceleration/deceleration in the seated occupant compresses the lumbar spine against the inertial load of the torso. Vertebral fracture occurs when compression tolerance of a vertebral structure is exceeded, with vertebral body fractures being the most common injury associated with vertical acceleration [18, 30, 38, 55]. From an injury biomechanics and safety engineering standpoint, it is important to understand the biomechanics of these high-rate axial acceleration-related fractures to prevent or lessen injury severity. In particular, orientation of the lumbar spine at the time of load application can change the load sharing pattern, alter injury characteristics, and affect injury tolerance [6, 9, 12].

Laboratory-based experimentation is the best method to characterize biomechanical injury tolerance and injury outcomes in specific loading environments. Human cadaveric lumbar spines, or appropriate biofidelic surrogates, are tested in controlled environments and the association between loading/boundary conditions and injury outcomes can be delineated. Whole segment models, such as the whole lumbar spine, are necessary to determine the effects of spinal orientation on injury characteristics. However, few studies have investigated whole lumbar column dynamic biomechanics, so the effects of geometrical parameters such as lordotic curvature and spinal tilt have not yet been determined under military-relevant acceleration scenarios. It is well known that normal lumbar spine lordotic curvature varies considerably between individuals (approximately 40–80 deg) [42] and can be significantly affected by seating position [24]. This range of spinal geometries based on personal variation and seating position at the time of loading can affect stress distribution and load transmission in the spine [5, 11, 21, 34], shifting injury location and characteristics from the anterior vertebral cortex to the posterior elements. For example, flexed postures demonstrated increased peak stress compared to normal postures [6, 11] and bias loads toward the anterior column. Given varying fracture tolerance and clinical significance for different spinal fracture types, it is extremely important to characterize the influence of spinal orientation on lumbar spine fracture outcomes during military-relevant loading situations.

The goal of this study was to explore how different lumbar spine orientations affect fracture characteristics. Sagittal and coronal orientations were quantified for lumbar spines prior to high-rate axial loading as experienced in military environments. Retrospective analysis correlated pre-test orientations with resulting fracture characteristics. The hypothesis was that orientations that bias loads to the anterior and middle columns of the spine will produce vertebral body fractures while orientations that result in greater extension moments will produce posterior element fractures. This study provides a novel approach to the field of lumbar spine injury biomechanics by using whole lumbar spines to explore the effects of spinal orientation at the time of accelerative load application.

## Methods

This study tested lumbar spines under high-rate vertical acceleration. Twenty-six lumbar spines were obtained from post-mortem human subject (PMHS) donors (12 female, 14 male), with an average age of 46.65 years (SD: 12.85). The protocol was approved by the Research and Development Committee and relevant subcommittees at the Zablocki Veterans Affairs Medical Center in Milwaukee, WI. Pre-test CT scans were used to ensure that the spines were without severe degeneration; all specimens had consistent alignment and were without bridging osteophytes or severe intervertebral disk degeneration. Included spines had an average intervertebral disk grade of 0.24 ± 0.53 (grade 0: normal disk; grade 1: mild degeneration; grade 2: moderate degeneration) [23].

The experimental protocol was previously published in Stemper et al [48]. Skin and extra-spinal musculature were removed; test specimens included only vertebrae, spinal ligaments, facet joints, and intervertebral disks. T12 and L5 were potted in polymethylmethacrylate (PMMA) to allow attachment to the drop tower. L5 was potted to include as much of the vertebral body and spinous process as possible without constraining the L4–L5 intervertebral disk and facet joints. Similarly, T12 was potted to include as much of the vertebral body and spinous process as possible without constraining the T12-L1 disk and facet joints. Each spine was potted with the L2-L3 disk horizontal for consistency in boundary conditions across all specimens [48]. Spines were allowed to maintain the curvature they naturally assumed and were not forced into any position. Due to differences in natural lordosis between specimens, each specimen had a unique curvature and orientation relative to the applied load on the T12 fixation.

**Fig. 1:**
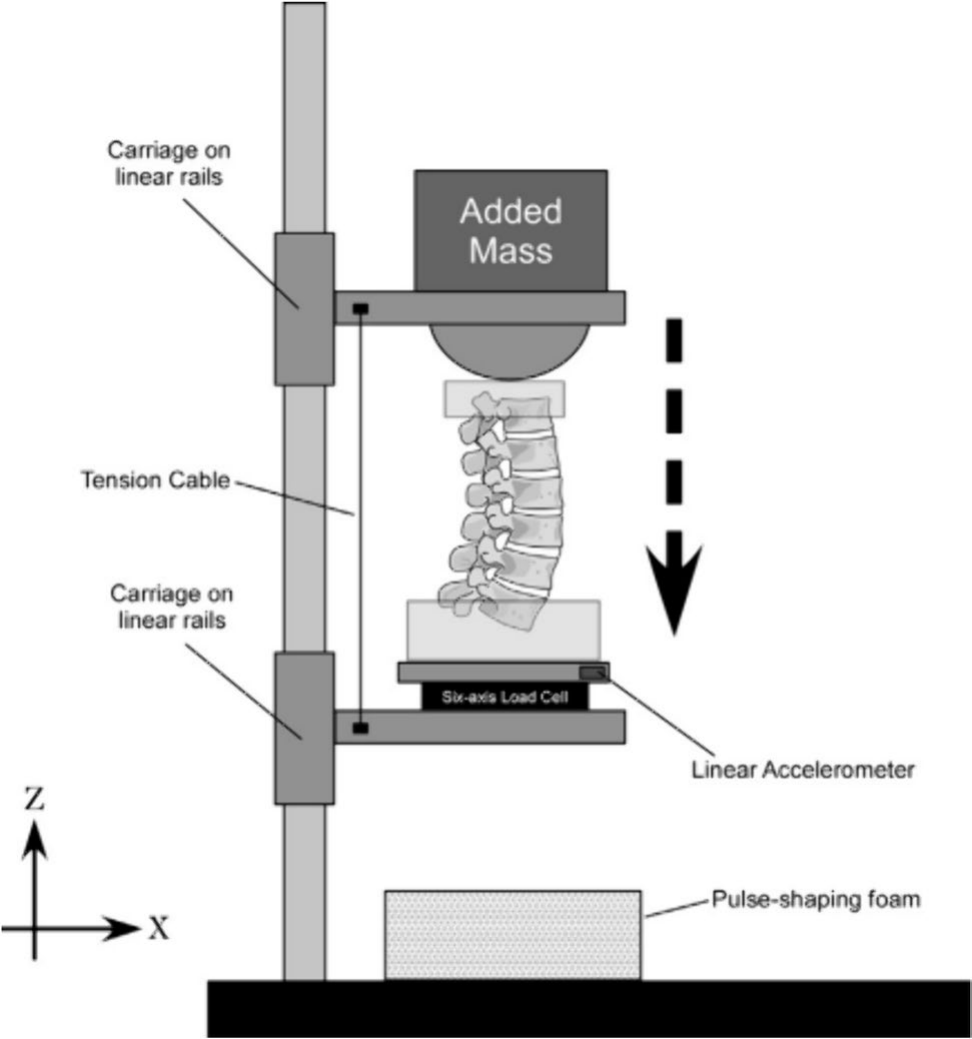
Experimental setup to simulate high-rate vertical acceleration of lumbar spines. Permission obtained from Stemper et al. J Biomech Eng 2011

The experimental setup consisted of two decoupled platforms attached to linear rails on a 7.6-m drop tower (Fig. 1). A 32 kg mass was added to the top platform to simulate the head–neck, torso, and upper extremities mass of a 50th percentile male [8]. Each specimen was pre-flexed with a 5 Nm moment for a consistent preload between specimens and to place them in their stiffest orientation [28]. Once pre-flexed, specimens were held in place by the loading cylinder (top platform) with the PMMA on the T12 vertebra. The bottom of the specimen was rigidly attached to the lower platform through a six-axis load cell. This was the pre-test orientation used for later measurements. A cable connected the two platforms to allow the upper platform to apply compressive loads to the cranial aspect of the specimen but prevent increased vertical displacement that would cause the loading cylinder to lose contact with the cranial specimen fixation during dynamic testing. X-rays were obtained prior to testing with each specimen in its pre-flexed orientation.

**Fig. 2:**
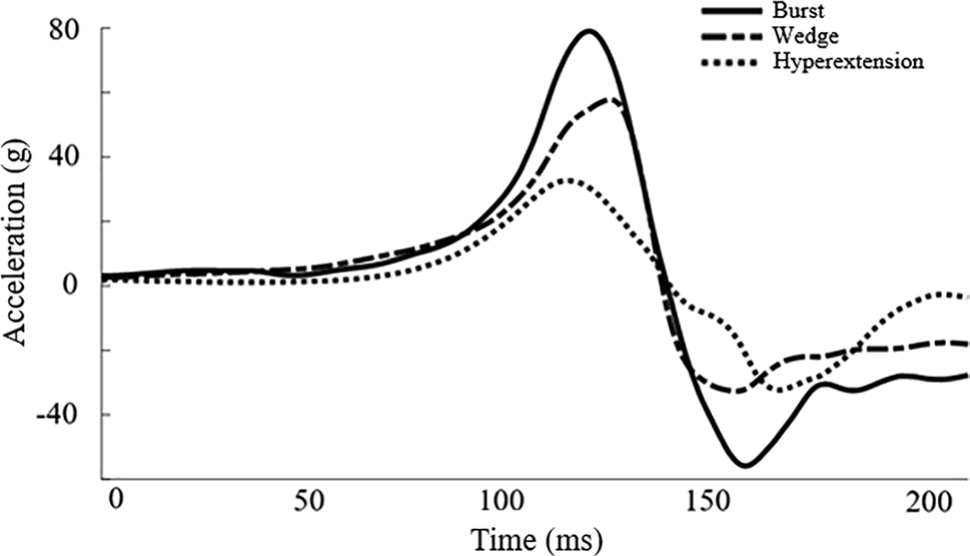
Average acceleration pulse for spines with burst (solid line), wedge (dashed line), and hyperextension (dotted line) fractures

The entire apparatus (spine, platforms, torso mass) was raised on the drop tower to a pre-determined height (ranging from 23 to 175 cm) based on the desired acceleration pulse designed to replicate high-rate axial loading experienced in military environments and held in place. At the initiation of the dynamic test, the entire apparatus was accelerated by gravity down the drop tower and decelerated at the base of the drop tower using a foam interface. Deceleration characteristics were controlled by the drop height and the mechanical properties of the pulse-shaping foam. Initial iterations of the acceleration pulse were focused on matching the rate of onset and peak acceleration of the naval aircrew common ejection seat (NACES) [48]. However, as the acceleration pulse was designed to safely remove the pilot from the aircraft in an emergency, acceleration pulses in the present study were increased in severity to produce lumbar spine fracture (Fig. 2). Vertical acceleration was measured using redundant accelerometers attached to the lower platform with a sampling rate of 20 kHz. Forces and moments of each test were recorded at the base of the specimen at 20 kHz using a six-axis load cell. Acceleration and inertially compensated force data were filtered using a low pass filter with a cutoff frequency of 1650 Hz. The sagittal plane bending moment was translated from the load cell to the center of segmental rotation at the mid-height of the L5 posterior vertebral body using pre-test X-rays and CTs. The horizontal distance between the center of the load cell and L5 was measured using ImageJ, and an appropriate couple was added to the sagittal plane bending moment. The peak acceleration, force, and moment were grouped based on fracture type to determine whether peak acceleration, force, or moment were dependent on fracture type.

Lateral X-rays were obtained following each dynamic test with the specimen in its post-test position to determine if bony fracture had occurred and detect any changes in spinal alignment or disk height. If no bony fractures were identified on X-ray, specimen palpation was performed at each spinal level and qualitatively compared to pre-test assessments to identify segmental soft tissue laxity that may have been associated with soft tissue injury. The same technician performed all palpation assessments. If no bony or soft tissue injuries were detected, additional dynamic tests with increased drop height were performed with each specimen following the previous dynamic test. Testing was stopped for each specimen once bony fracture or soft tissue injury was identified. Post-test CT scans were then obtained for all spines. Video analysis was used to confirm primary fracture types. These primary injuries were included in the classification of injury type and any secondary injuries that occurred in the same spine were not included.

Vertebral fractures were classified using the AOSpine thoracolumbar classification system [52]. The AOSpine classification system categorizes vertebral fractures into three groups: compression injuries, distraction injuries, and displacement/translational injuries. Compression injuries involve the vertebral body, with wedge compression fractures involving fracture of only the anterior cortex and burst compression fractures involving fracture of one or both endplates. Hyperextension fractures involved fracture of the posterior elements with concomitant anterior longitudinal ligament rupture.

**Fig. 3:**
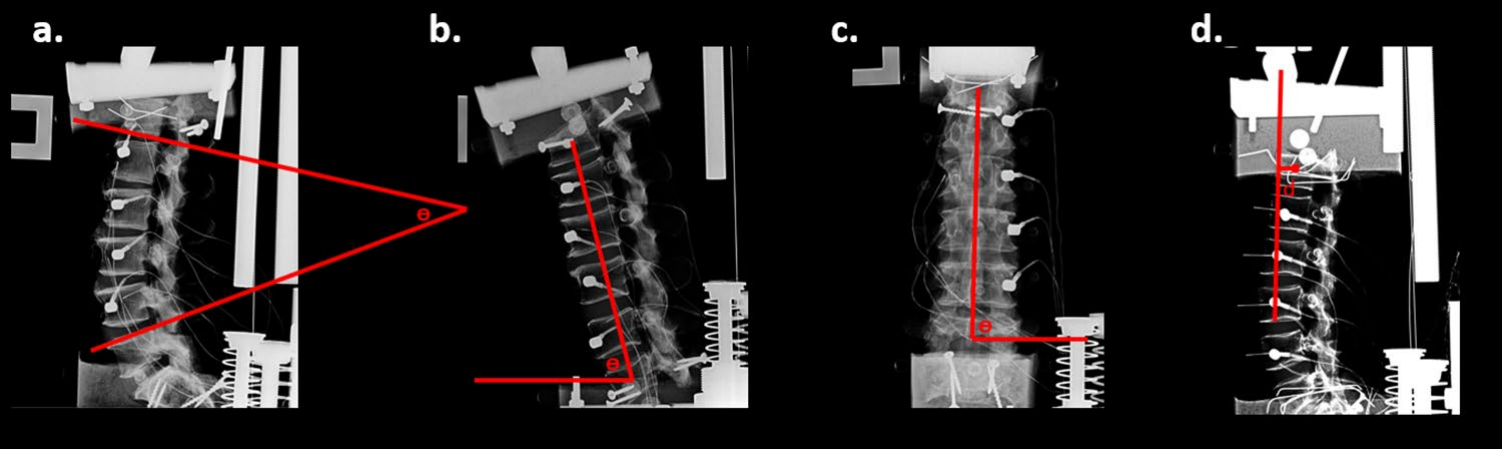
Measurements of pre-test spine orientation made using ImageJ. **a** Cobb angle, **b** Sagittal lumbar spine angle with the horizontal, **c** Coronal lumbar spine angle with the horizontal, and **d** Location of the load relative to the centroid of the vertebral body of T12

Measurements of sagittal and coronal spinal orientation were completed using ImageJ (Fig. 3). The Cobb angle was measured as the angle created by the intersection of two lines extended posterior along the inferior aspect of the T12 vertebral body and the superior aspect of the L5 vertebral body. The sagittal and coronal tilts of the spine were quantified by measuring the angle of a line drawn through the vertebral body centroids of T12 and L5 from horizontal. The location of the load on T12 was found by measuring the horizontal distance between the application of the load (i.e., center of the loading cylinder from the upper platform) and the vertebral body centroid of T12. The vertebral body centroid was found by drawing diagonal lines to connect opposite vertebral body corners, forming an x across the center of the vertebral body. The center of the x was used as the centroid.

Primary measurements were completed by the first author R.C. All measurements were then repeated by the first author R.C., as well as the second and third authors M.K. and N.S., who each completed half of the measurements. Interrater and intra-rater reliabilities were calculated using Pearson’s correlation coefficient. All statistical analysis was completed in RStudio 2023.06.2. Non-parametric statistics were used due to the small sample size. Experimental parameters and pre-test spinal orientation measurements were compared for fracture types using Kruskal–Wallis rank sum tests with a significance level of *p* < 0.05. Wilcoxon rank sum tests were used to determine significant pairwise differences in fracture type for all metrics that had a significant main effect of fracture type.

## Results

**Fig. 4:**
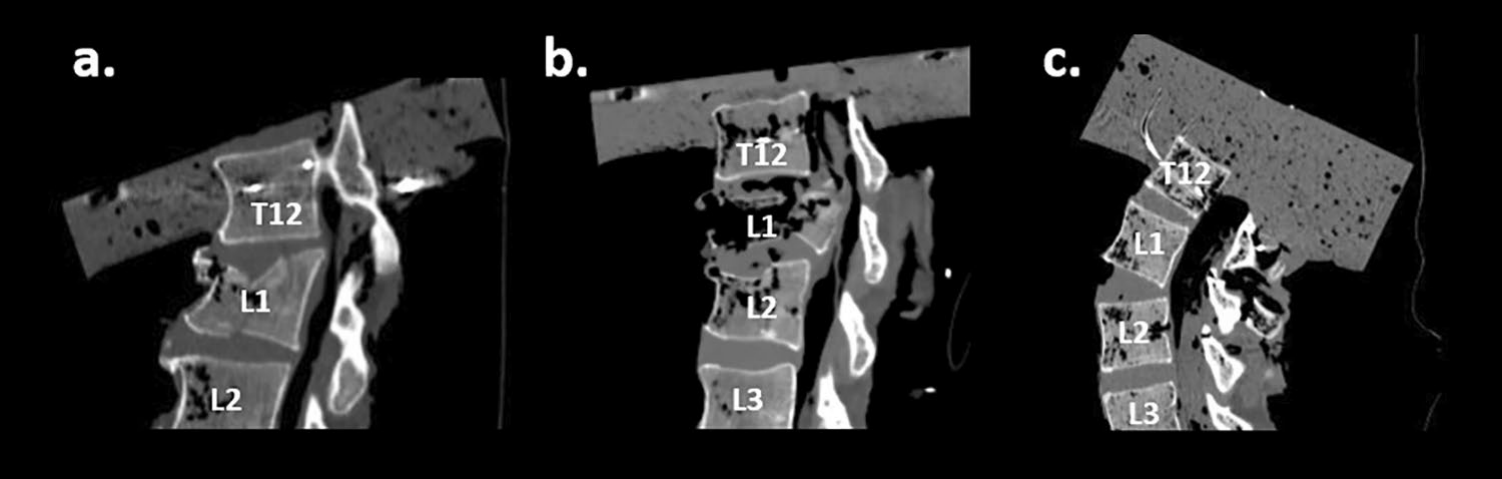
Examples of **a** wedge, **b** burst, and **c** hyperextension fractures from post-test CT scans

A total of 26 lumbar spines were tested. Three spines experienced segmental laxity without vertebral fracture following a dynamic test. Testing was stopped for those three spines which were excluded from this analysis. Two spines sustained spinous process fracture, and one spine sustained hyperflexion injury. Those three spines were also excluded from this analysis, due to the small sample size that prevented statistical comparison to the other groups. Six of the spines failed at the fixation sites (T12/L5) and were also excluded from this analysis. The remaining 14 spines (five female/nine male) that sustained wedge, burst, and hyperextension fractures were included in this analysis (Table 1). The specimen donors had an average (SD) height of 169.1 (14.6) cm and weight of 71.0 (16.6) kg. Due to the small sample size, no additional analyses were performed to determine potential differences in fractured spinal level. Examples of CT scans demonstrating each fracture type are provided in Fig. 4.

**Table 1:** Fracture locations and pre-test spine orientations for each specimen

Fracture type	Sex	Fracture location	Cobb angle (degrees)	Spinal tilt (degrees)	Load location (cm)
Burst	F	L3	14.8	78.4	–
Burst	M	L1	4.8	76.2	0.54
Burst	F	L1	6.7	71.8	0.38
Burst	M	L1	6.8	76.8	0.47
Hyperextension	M	L2	18.2	–	0.19
Hyperextension	M	L1	34.6	70.1	0.46
Hyperextension	F	L2	55.4	71.7	0.35
Hyperextension	M	L3	26.8	81.6	–
Wedge	M	L1	10.6	80.5	0.81
Wedge	M	L1	18.1	–	0.65
Wedge	F	L1	6.6	87.9	0.49
Wedge	M	L1	20.6	–	0.41
Wedge	M	L1	30.3	89.1	0.38
Wedge	F	L1	39.1	78.8	0.57

Of the 14 spines included in analysis, four spines had multiple fractures following the dynamic test. Each spine had one compression fracture (wedge/burst) and other injuries that were more likely associated with shear or bending as the lumbar column buckled. Video analysis confirmed that the primary fracture for all four spines was the compression fracture, and the shear/bending fractures were a result of column instability and continued loading of the unstable spine. Only primary fractures were included in this analysis.

The interrater correlation coefficient was *r* = 0.894, and the intra-rater correlation coefficient was *r* = 0.975 for pre-test orientation measurements (Cobb angle, spinal tilt, load location). Three spines were excluded from the spinal tilt measurement, and two spines were excluded from the load location measurements due to inadequate visualization of the necessary measurement points on the pre-test X-ray. The 14 spines included in this analysis had an average age of 41.6 years (SD: 14.4). Two spines had more than one dynamic test. One spine had two dynamic tests and one spine had three dynamic tests. All spines with multiple dynamic tests sustained wedge fractures. The spine with two dynamic tests was dropped from heights of 40 cm and 127 cm, fracturing following the 127 cm drop. The spine with three dynamic tests was dropped from heights between 40 and 46 cm, fracturing following the 46 cm drop. The average difference in Cobb angle between the dynamic non-injurious tests and the dynamic injurious tests for each spine was 3.1 (3.7) degrees. There were no trends in Cobb angle changes in subsequent tests with the same specimen.

Peak failure acceleration and force were similar for spines with one dynamic test compared to spines with multiple dynamic tests. Peak force and acceleration were similar across fracture types (*p* = 0.44; *p* = 0.28). Peak average (SD) acceleration, force, and bending moment can be found in Table 2.

**Table 2:** Peak failure acceleration, force, and sagittal plane bending moment

	Mean (SD)		
	Peak acceleration (g)	Peak force (N)	Peak sagittal moment (Nm)
Wedge	57.0 (32.4)	6668 (2260)	− 50.5 (166.8)
Burst	76.7 (60.1)	6406 (1040)	− 218.0 (70.1)
Hyperextension	30.5 (11.1)	5239 (1049)	306.0 (166.8)

Fracture type was dependent on peak sagittal plane bending moment at the specimen base (*p* = 0.009). Pairwise comparisons revealed that spines with a hyperextension fracture experienced a larger peak sagittal plane bending moment compared to spines with wedge (*p* = 0.04) and burst (*p* = 0.04) fractures.

Fracture type was nearly dependent on Cobb angle (*p* = 0.07) (Table 3). Pairwise comparisons revealed that spines that sustained hyperextension fractures had larger Cobb angles than spines that sustained burst fractures (*p* = 0.09). Average Cobb angles of spines that sustained hyperextension fractures were 67.1% and 29.3% greater than Cobb angles for spines that sustained burst and wedge fractures.

**Table 3:** Average pre-test spinal orientation measurements

	Mean (SD)			
	Lordosis	Tilt		Load
	Cobb angle (degrees)	Lumbar spine angle sagittal (degrees)	Lumbar spine angle coronal (degrees)	Location of load on T12 (cm)
Wedge	20.9 (12.2)	84.1 (5.2)	88.7 (1.6)	0.55 (0.16)
Burst	8.3 (4.4)	75.8 (2.8)	90.1 (1.9)	0.47 (0.16)
Hyperextension	33.7 (15.9)	74.4 (6.2)	90.1 (2.4)	0.33 (0.14)

Fracture type was nearly dependent on sagittal lumbar spine angle (*p* = 0.07) (Table 3). Spines that sustained wedge fractures were more upright compared to spines that sustained burst fractures (*p* = 0.09). Spines that sustained burst or hyperextension fractures were tilted an average of 8.2° and 9.6° further anteriorly than spines that sustained wedge fractures. Fracture type was not dependent on coronal lumbar spine angle (*p* = 0.74, Table 3).

Fracture type was not significantly dependent on anterior–posterior location of the applied load in relation to the T12 centroid (*p* = 0.13) (Table 3). In general, spines that sustained wedge fractures had the load placed more anteriorly relative to the T12 vertebral body centroid compared to spines that sustained burst (15.3%) and hyperextension (39.3%) fractures.

## Discussion

The objective of this study was to determine the relationship between lumbar spine orientation and fracture characteristics resulting from dynamic compressive loads. This unique study incorporated a whole lumbar column laboratory-based model that reproduced dynamic compressive loading using a biofidelic setup that inertially loaded the cranial end of the lumbar spine while decelerating the base [48]. The most common type of injury was a vertebral body injury, which is representative of the lumbar injuries that occur in military environments such as helicopter crashes, aircraft ejections, and under-body blasts [30, 38].

The original hypothesis was that spinal orientation at the time of loading would affect fracture characteristics by biasing the load to different spinal elements. Each spine was allowed to maintain its natural curvature during preparation, then pre-flexed with a 5 Nm flexion moment for consistent boundary conditions. The range of tested lumbar spine angles was within the range of the general population [42]. Results of this study supported the hypothesis that spines with greater lordotic curvature sustained injury to the posterior elements as the convex curvature biased load transmission to the dorsal spine. Straighter spines sustained vertebral body fractures as the compressive load path was more in line with the vertebral bodies and disks. Spinal tilt and the location of the load vector also influenced fracture type. Anteriorly tilted spines with a centrally applied load sustained burst compression fractures, as the load was applied evenly across the intervertebral disk, resulting in an endplate fracture. Vertically oriented spines with an anteriorly applied load sustained wedge compression fractures, as the load was applied in the anterior column of the spine, biasing the spine toward an anterior column fracture. These important findings demonstrate for the first time in a whole-column laboratory-based model the importance of spinal orientation on fracture characteristics in the lumbar spine.

Force tolerance of the lumbar spine under axial loading was studied using various models including isolated vertebrae, two- and three-vertebrae segments, and complete lumbar spines. These models resulted in different fracture tolerances. Individual lumbar vertebrae had peak failure forces of approximately 10 kN [35, 49]. Two-vertebrae segments (i.e., functional spinal units) had similar peak failure forces to isolated vertebral bodies at between 11 and 13 kN [12]. Failure forces were considerably reduced for three-vertebrae segments with reported peak values of 6–7 kN [36, 44, 56]. Whole lumbar spine columns were similar to three-vertebrae segments with a tolerance of 5–6 kN [12, 47]. Accordingly, lumbar spine tolerance is different between isolated vertebrae/short segments and longer segments (i.e., 3 + vertebrae), which highlights the role of the intervertebral disk in the biomechanics of lumbar spine vertebral fracture. Compressive loading of isolated vertebrae involves the load-carrying capacity of the cortex and fracture tolerance is related to cortical failure. This mechanism is consistent with anterior wedge fractures produced in the current study, although wedge fractures concentrate stress in the anterior cortex and isolated vertebral body testing distributed loads across the entire axial plane of the vertebral body which may explain lower wedge fracture forces in this study. Compression of 3 + vertebral body segments involves interaction between the intervertebral disk and endplate, and fracture tolerance is associated with endplate fracture as the nucleus overloads the weaker central region [17] of the endplate to produce burst fracture [9]. Whole lumbar spine fracture biomechanics, as reported in this manuscript, includes geometrical effects of the column that incorporate increased bending moments and affect compressive load transmission and load sharing between different components and levels of the spine. Accordingly, as shown in this study, lumbar spine fracture presentation was dependent on spinal orientation at the time of loading as it biased prestress and load transmission to different structures in the spine. From an injury prevention perspective, some spinal orientations may be more protective than others.

The novelty of this study lies in the use of whole-column PMHS lumbar spines to determine the effects of spinal orientation at the time of accelerative loading on resulting compressive fracture characteristics. Whole-body PMHS were previously used to study lumbar spine fracture biomechanics in vertical accelerative environments, specifically in under-body blast [10, 45]. However, whole-body PMHS do not offer the same level of experimental control regarding load application and spinal orientation, nor the same level of detail in biomechanical outputs, as component testing. Therefore, whole-body PMHS are not ideal for studying the mechanisms by which spinal orientation affect lumbar spine fracture characteristics. Finite element models are also commonly used to simulate the lumbar spine under various loads [11, 27, 46, 54, 60]. However, simplified anatomical detail precludes the examination of differing injury mechanisms/characteristics. For example, contemporary finite element models typically include simplified endplate/vertebral body characteristics that make it difficult to differentiate between wedge and burst fractures. Beyond the studies resulting from our test series [41, 47–49], the WIAMan project was the only other study to incorporate accelerative loading of whole lumbar columns to study the biomechanics of lumbar spine fracture [57–59]. That study was not focused on determining the effects of spinal orientation on fracture type and tested all specimens in an identical pre-determined orientation regardless of the natural curvature and stiffness of each lumbar column. Other whole-column studies incorporated pistons and weight drop apparatuses to impact the cranial aspect of the stationary lumbar spine [12, 14, 32, 44]. As such, the present study is unique in its focus on characterizing the effects of spinal orientation on fracture characteristics produced during vertical accelerative loading.

The effects of orientation on injury outcomes was previously investigated to some degree using shorter segments. Boisclair and colleagues investigated the effect of spine orientation and demonstrated that two-level functional spinal units (consisting of three vertebral bodies and two intervertebral disks) with 15° flexion sustained anterior compression fractures, whereas specimens with 0° flexion sustained burst fractures [2]. Curry and colleagues reported single-level spinal units (two vertebral bodies and one intervertebral disk) only sustained endplate fracture when quasi-statically loaded in compression with adjacent endplates oriented in parallel [9]. Although Curry and colleagues did not produce vertebral body burst fracture, endplate fracture is known to precipitate burst fracture as dynamic endplate failure leads to disk tissue being driven into the vertebral body resulting in outward burst fracture of the surrounding cortex [39, 49]. Therefore, experimental data from quasi-static testing of functional spinal units reported by both Boisclair and Curry support the current dynamic whole-column findings on the influence of spinal orientation on fracture characteristics.

These findings are relevant for high-rate acceleration environments that can be experienced by military personnel. Aircraft ejections, helicopter crashes, and under-body blasts all place the lumbar spine under vertical accelerations that produce compressive loads and carry a risk of lumbar spine fracture. Seated orientation of the lumbar spine differs between different aircraft platforms and individuals may accommodate differently based on anthropometry, natural spine orientation, and control. For example, the F-16 seat is reclined 30° relative to the vertical along with the control stick positioned on the right-hand side of the cockpit. The F-15 seat is more vertical with a center control stick [20, 43]. Fighter pilots also actively move their bodies during combat to track opponents. Helicopter pilots change their torso position from forward to vertical to reclined based on position within the aircraft and type of flight [4]. Individuals in the gunner’s position sit vertically while those in the pilot’s seat lean forward and to the left, placing their right elbow on their right thigh in a position typically known as “helo-hunch” [15]. The present study has shown that these different body positions during flight may have a dramatic effect on injury characteristics and associated clinical outcomes.

In ground-based vehicles, a number of factors affect seating position for drivers and crew members. For example, stature, body-borne equipment, and vehicle seat design can dramatically alter the seating position of the occupant, and therefore the lumbar spine orientation. For example, personal protective equipment such as helmets and tactical vests can affect natural sitting position [40]. Understanding the role of lumbar spine orientation in the tolerance and outcomes of vertebral fractures can be used by vehicle designers and safety engineers to design safer environments and provide safety recommendations for military personnel.

From a clinical standpoint, spinal orientation at the time of injury is vital to the clinical presentation and patient outcomes. Proper management of spinal fracture is essential as a delayed or missed diagnosis can result in chronic pain and neurologic injury [26]. Lumbar spine stability is classified according to the three-column concept. The anterior column is composed of the anterior longitudinal ligament, the annulus fibrosus, and the anterior half of the vertebral body. The middle column consists of the posterior longitudinal ligament, the posterior annulus fibrosus, and the posterior half of the vertebral body. The posterior column includes the posterior elements from the pedicles through the supraspinal ligament, including the facet joints.

Thoracolumbar fractures are typically classified using Thoracolumbar Injury Classification and Severity Score (TLICS) and the Thoracolumbar AOSpine Injury Score (TL AOSIS) as the latter was used in this project [52]. Moreover, lumbar spine fractures are outcomes of three primary mechanisms of injury: axial compression, axial distraction, and translation that give rise to four fundamental patterns of injuries: wedge compression fractures; stable and unstable burst fractures; flexion-distraction injuries; and translational injuries [7]. Clinically the spine is determined to be stable or unstable based upon the integrity of two of the three spinal columns [33], with the middle column deemed to be the major determinant of spinal stability [37].

Fifty to 70% of all civilian thoracolumbar fractures are wedge compression fractures [19]. These types of fractures are commonly observed in the elderly, especially those at increased risk of osteoporosis or osteopenia [53] or in the setting of blunt trauma [19]. Typically, these are caused by anterior column insufficiency due to an axial load applied in flexion. Compression fractures that involve solely the anterior column and do not interrupt the posterior longitudinal ligament or posterior vertebral body cortex are considered stable. Importantly, an unstable fracture may be created from a compression fracture, acute decrease in vertebral height by 50% or more, or if there is additional involvement of a rotational force greater than 20° fracture kyphosis, or multi-vertebral compression fractures as all of these may lead to posterior ligament and/or middle column involvement, thus giving rise to spinal instability and possible neurological injury [1]. Moreover, Holmes and colleagues concluded that 14% of thoracolumbar injuries are burst fractures in the setting of blunt trauma [19]. These are caused by forces that disrupt the vertebral endplate, thus all burst fractures are deemed unstable as 42–58% of patients experience additional neurological injuries [19]. Burst fracture can occur with or without posterior column involvement, which significantly increases the risk of neurological injuries [16].

Flexion-distraction injuries account for 10% of all thoracolumbar spinal column injuries as forces lead to anterior and middle column involvement as well as a tear in the posterior longitudinal ligament. Although flexion-distraction injuries can involve the anterior and middle column, 10–25% of flexion-distraction injuries can have pure ligamentous involvement [16]. Isolated transverse process fractures, fractures of the pars interarticularis, laminar fractures, spinous process fractures, or facet fractures are deemed to be minor fractures and account for 14% of injuries. In fact, most minor spinal injuries occur in the lumbar region and are considered stable. These fractures become of concern when there is high velocity trauma involved that can cause potential lumbosacral plexus injury [22].

Importantly, clinical management of spinal column injuries is typically based on stability of the fracture pattern. The first stage of clinical management is to determine the presence/absence of spinal instability or severe neurologic abnormalities through physician evaluation or imaging. If there is spinal instability present (e.g., spinal burst fracture), the patient must be admitted for urgent evaluation by a neurosurgeon [31]. These patients typically require immediate decompression and stabilization though there is no definitive method to achieve this as physicians may use closed reduction using traction or open surgical procedures [13]. On the other hand, if the fracture is deemed to be stable (e.g., anterior wedge fracture) and there is no evidence of neurological deficit, outpatient management is possible. Outpatient management usually includes pain control through analgesics and follow-up monitoring.

This study retrospectively analyzed the influence of orientation prior to high-rate vertical acceleration on lumbar spine fracture type. While each injury group included at least four specimens, this is still a small sample size. Several additional variables, such as sex and age, were not able to be analyzed due to the small sample size. Sex has been shown to affect vertebral body failure characteristics, due to geometric differences in vertebral body size [3, 49]. Males and females also exhibit differences in spinal curvature [29, 51]. Female lumbar spines have greater lordosis, which may increase risk of hyperextension injuries. Male lumbar spines are straighter, which may shift stress anteriorly and increase the risk for vertebral body fractures. This was not apparent in this study, as there were no trends for spinal orientation between males and females.

Age has also been associated with lumbar lordosis, with older individuals having larger lordosis [24, 51]. Increased lordosis associated with older age may increase the risk of lumbar spine hyperextension injuries. In this analysis there was no obvious relationship between fracture type and age (mean age (yrs): wedge: 40, burst: 55, hyperextension: 41). This could be due to the relatively young nature of the spines included in this study, as age-related increases in lordosis may occur at an older age.

This study was limited by a relatively small sample size that prohibited deeper analysis on the effects of male/female sex on injury outcomes/tolerance, and input parameters that affected injured spinal level. Another possible limitation was that the same 32 kg ‘torso’ mass was used for all specimens regardless of donor body size. In vivo, these spines may have borne a larger or smaller load compared to these experimental end conditions, which may have had an undetermined effect on injury metrics reported in this manuscript. However, the authors chose to maintain a constant ‘torso’ mass across all specimens tested in this study to reduce the number of variables and strengthen the statistical analysis.

Results from this study provided unique insight into the understanding of lumbar spine injury biomechanics by showing that spine orientation affected fracture characteristics in a dramatic and predictable manner. Specifically, under the same compressive loading conditions, lumbar spine curvature and orientation relative to the applied compressive loading vector shifted the fracture location from the anterior vertebral body for straightened and flexed spines to the posterior elements for spines maintaining greater lordotic curvatures. From an injury prevention standpoint, these findings lay the groundwork for understanding trade-offs that may be associated with different occupant positions during dynamic injury-producing events for both civilian and military environments. Other factors are identified, which require further investigation due to their influence on lumbar spine orientation and associated injury risk.
